# Examining the mental health symptoms of neurodivergent individuals across demographic and identity factors: a quantitative analysis

**DOI:** 10.3389/fpsyg.2025.1499390

**Published:** 2025-03-19

**Authors:** Elizabeth Kroll, Megan Lederman, Jonathan Kohlmeier, Jaime Ballard, Izabella Zant, Caroline Fenkel

**Affiliations:** ^1^Charlie Health, Inc., Bozeman, MT, United States; ^2^The Center for Applied Research and Educational Improvement, University of Minnesota, Saint Paul, MN, United States

**Keywords:** anxiety, depression, neurodivergence, ADHD, autism, mental health treatment

## Abstract

**Background:**

New research indicates that neurodivergent individuals experience unique intersections of identities at a much higher rate than historical research suggested. Factors such as race, gender, and sexuality interact with neurodivergent identities to influence their lived experiences and healthcare needs. While intersectionality has gained traction in research, the intersection of neurodivergence with other marginalized identities remains underexplored.

**Objective:**

This study seeks to fill this gap by examining how various social identities interact with neurodivergence to mediate mental health symptoms. Understanding these interactions is crucial for developing inclusive mental healthcare practices that affirm neurodivergent identities and address disparities in mental health outcomes among marginalized populations.

**Methods:**

Data was collected between May 2023 and March 2024 from 14,219 individuals admitted to a virtual intensive outpatient mental health treatment program. Clients self-reported demographic information, including neurodivergent identity, gender, sexual orientation, and race. Two-way MANOVAs were run to assess the interactions between different identities and the impact that those interactions had on anxiety and depression scores at both intake and discharge. When MANOVAs indicated significant interactions, follow-up two-way ANOVAs were conducted for each dependent variable.

**Results:**

Sexual and gender minority respondents were more likely to identify as neurodivergent compared to their straight and gender binary counterparts. Significant interactions were found between neurodivergence and gender on depression, as well as neurodivergence and sexual orientation on both depression scores and anxiety scores. However, no significant interactions were found between neurodivergence and racial identity with respect to depression or anxiety scores.

**Discussion:**

The complex interplay between neurodivergent identity and additional marginalized identities has a significant impact on how mental health symptoms are experienced, and therefore should have a significant impact on how treatment is tailored to the individual. By providing identity-affirming care, individuals are given the space to process their mental health symptoms in an empowering and less stressful environment.

## 1 Introduction

Neurodivergent individuals experience a rich and complex intersection of identities (Cooper et al., 2018; Dewinter et al., 2017; Fombonne and Zuckerman, 2022). Factors such as race, gender, and sexuality interact with their neurodivergent identities to impact their quality of life through the way that the world interacts with them and vice versa. Despite the growing recognition of intersectionality and neurodiversity in academic and social discourse, there remains a notable gap in research that explores the overlap of these two frameworks. This paper seeks to address this gap by examining mental health symptoms of neurodivergent individuals within the context of intersectionality. By expounding upon the ways in which various social identities intersect with neurodivergence and potentially mediate mental health symptoms, we aim to contribute to a deeper understanding of the lived experiences of neurodivergent individuals and advocate for more inclusive and equitable mental health treatment.

### 1.1 Identity formation and intersectionality

While identity development has been a subject of study since Erik Erikson introduced it as the fifth stage of development in the 1960s (Erikson, 1966), the exploration of how multiple identities interact and mediate individuals’ lived experiences has gained increased scholarly attention more recently. With the emergence of intersectionality theory. Introduced in the late 1980s by legal scholar Crenshaw (2013), intersectionality aims to shed light on the ways that coexisting identities may simultaneously impact a person’s lived experience within a social and political setting. The converse of intersectionality–the single determinant model of identity–proposes that one aspect of a person’s identity is the sole determinant of disenfranchisement within a system. Alternatively, intersectionality proposes that social identities have a complex and overlapping impact on privilege or disenfranchisement. For example, while Black men and women may share a racial identity, the ways in which they experience society differ due to their differences in gender identity (Gibson et al., 2016). Similarly, LGBTQIA+ people of color may experience compound discrimination relative to their white counterparts (Kisler, 2013). Combinations of marginalized identities effectively alter the experiences of individuals.

Intersectionality has recently gained traction in quantitative research, with a surge in quantitative studies post-2010 (Bauer et al., 2021). With the more recent adoption of this framework into quantitative research, many new studies have demonstrated important differences in healthcare quality based on the held identities of patients. For example, Morrow et al. (2020) interviewed Asian men about their mental health. Their direct quotes revealed that they were cognizant that mental health stigma cannot exist outside of the social frameworks of their gender and race. Similarly, Seng et al. (2012) conducted a secondary analysis on women with PTSD and found that the number of marginalized identities the women held significantly impacted PTSD symptoms and quality of life scores. Lastly, Mereish and Bradford (2014) found that sexual minority clients had a greater risk of lifetime substance use problems, with nuanced differences when considering gender and racial differences.

### 1.2 Neurodivergence

A burgeoning focus on neurodiversity in society underscores the potential utility of incorporating neurodivergent identities into intersectionality frameworks. Neurodiversity asserts that variations in cognitive processing are natural and not inherently negative. While we all exhibit neurodiversity between one another, neurodivergent conditions are generally thought of as diagnosable differences in neurocognitive processing. Conditions such as autism spectrum disorder (ASD) and attention deficit hyperactivity disorder (ADHD) fall under the umbrella of neurodivergence, along with other neurocognitive and developmental conditions. Additionally, as the neurodivergent movement continues to grow and evolve, there are increasing calls for neurodivergence to include mental health disorders such as anxiety or OCD due to the changes in cognitive processing that are common in these disorders. As this paper’s focus is about the impact of identity on mental health symptoms, anyone who identifies as neurodivergent, regardless of clinical diagnosis, was considered for the sake of analysis.

Individuals who identify as neurodivergent frequently report worse depression, anxiety, and quality of life scores (Kroll et al., 2024; Lai et al., 2019). Additionally, they tend to have higher utilization rates in healthcare settings (Zerbo et al., 2019; Gupte-Singh et al., 2017), but worse quality of life outcomes (Fleming et al., 2017; Loe and Feldman, 2007; Van der Oord et al., 2005). The current state of healthcare for neurodivergent individuals clearly indicates that our understanding of neurodivergent needs is severely lacking. Following the disability movement, the neurodivergent paradigm posits that it is not an individual’s deficits that impose hardship, but rather society’s lack of accommodations. In other words, it is a neurodivergent individual’s marginalization that contributes to their disenfranchisement, not their neurodivergence directly.

This lack of understanding may be driven by the fact that historically, neurodivergent research has focused primarily on white males in clinical settings (Kalb, et.al., 2022). As a result, this research has left out a large portion of the neurodivergent population (Craddock, 2024; Brickhill et al., 2023; Lovelace et al., 2021). However, research has shown that individuals who identify as neurodivergent are more likely to hold other marginalized identities, such as gender and sexual minority identities (Cooper et al., 2018; Dewinter et al., 2017), who are already at risk of developing severe mental health conditions (Berry et al., 2023; Robertson et al., 2021). This finding lends additional credence to the argument that neurodivergence must be viewed within the lens of intersectionality, as a majority of neurodivergent individuals are not white males, as the historical research suggested (Krahn and Fenton, 2012).

### 1.3 Intersectionality in neurodivergent-affirming care

Since the majority of neurodivergent research has been conducted with white males (Kalb, et.al., 2022), individuals of other races and genders tend to be underdiagnosed (Bennett and Goodall, 2022) and under-supported (Dababnah et al., 2018). Further research is required to understand the needs of these underserved populations. Additionally, because autism was initially viewed, and is often still categorized, as a social deficit. Consequently, autistic individuals were often thought of as lacking a rich social life and social identity. As we grow to understand neurodivergence as a social identity and culture, it is imperative that we begin investigating the impact it has on individuals who may not fit the historic model of neurodivergence.

Identity-affirming care, which acknowledges and accepts clients’ identities, is rooted in the theory of the social structure of health, or the idea that the conditions in which people live, learn, work, play, worship, and more, affect quality of life and health outcomes. While studies have shown that affirming neurodivergent identities is associated with better clinical outcomes (Kroll et al., 2024; Davies et al., 2024), further exploration is needed to understand how this acknowledgement may vary based on intersecting identities (Botha and Gillespie-Lynch, 2022). Until neurodiversity is viewed under an intersectional lens, we cannot provide the best treatment to neurodivergent individuals because we do not fully understand how their other identities interact with their neurodivergence and ultimately impact their lives. Incorporating intersectionality into therapy practice, as proposed by Chantler (2005), could lead to more equitable mental health outcomes by addressing disparities faced by marginalized groups.

### 1.4 Study objectives

This paper aims to identify common intersections and interactions between neurodivergent identities and other major marginalized identities. The first objective of this paper is to analyze the rate at which neurodivergent clients within a high acuity population hold additional marginalized identities. The second objective is to assess the interaction between neurodivergent identity and mental health symptoms when a client holds other marginalized identities. These findings will then be used to make clinical recommendations for the treatment of marginalized individuals who also hold a neurodivergent identity.

## 2 Methods

### 2.1 Setting

Data collection took place at Charlie Health, a virtual intensive mental health treatment program that primarily worked with high acuity individuals between the ages of 11–35 at the time of this study. The majority of clients present with high acuity mental health concerns such as suicidal ideation, self-harm, high depression scores, or high anxiety scores. Charlie Health’s programming consists of 9 h of group sessions, 1 h of individual therapy, and an optional hour of family therapy per week, plus psychiatry as-needed. An integral part of Charlie Health’s treatment model is a compassion-focused and identity-affirming approach to care. Each group session is easily adaptable to create inclusive and supportive environments. These adaptations include addressing sensory needs, allowing for multiple dimensions of communication, and an exploration of social identity.

### 2.2 Ethics statement

This study was reviewed and approved by the NorthStar Institutional Review Board (IRB), who deemed this investigation exempt as secondary research usage (NB400214).

### 2.3 Statement on positionality and participatory research

The lead researcher and author of this paper identifies as a neurodivergent woman and recognizes the differences in symptom presentations between demographic groups within the course of mental health treatment. Her goal is to highlight the ways in which these differences can be affirmed and utilized in treatment settings to provide personalized care to neurodivergent individuals. Their background is informed by quality improvement work in a mental health setting and additional identity research.

Furthermore, the questions posed regarding neurodivergence were written in conjunction with Charlie Health’s Director of Clinical Curriculum who identifies as neurodivergent. Questions regarding race and sexual orientation were reviewed and approved by Charlie Health’s Director of DEI. All analyses were reviewed by multiple individuals who identify as neurodivergent, as well as individuals who do not identify as neurodivergent but have a history of working with neurodivergent clients.

### 2.4 Data collection

Between May 2023 and March 2024, intake data were collected from 14,219 participants. Clients were given an intake survey in the first hour of their orientation to group therapy. A Charlie Health staff member joined the group and distributed personalized links to each client. Staff members stayed in the group to answer questions until all clients were finished. Clients were instructed that the survey was optional and would not affect their admission status.

Of those 14,219 participants, 9,782 submitted discharge surveys. Discharge surveys were distributed on the client’s last day in group therapy. Clients were pulled into a breakout room on Zoom with a Charlie Health staff member who then gave the client a personalized link to their discharge survey. Clients were informed that the survey was optional and would not affect their discharge status. If the client opted out of the survey, they were sent back to their group; otherwise, once the survey was complete, they were sent back to their group. If a client missed their final group session, they were emailed and texted a personalized link to the discharge survey and prompted to fill it out with a $25 incentive.

### 2.5 Measures

#### 2.5.1 Demographics

Demographics were collected at intake. Clients were asked to disclose their age, gender, sexual orientation, race, and neurodivergent identity. For this study, a neurodivergent identity of “other” could be anything the client deemed neurodivergent. This is due to the lack of a universally agreed-upon social definition for neurodivergence. All questions were multi-select to allow clients to select all answers that they felt represented them.

**Table:** 

Demographics category	Answer options
Gender	Male Female Non-binary Genderqueer Nonconforming Gender fluid Gender Neutral Other
Sexual orientation	Straight Asexual or Gray-sexual Bisexual Pansexual Gay Lesbian Queer Questioning Other
Race	Black or African American Indigenous People Around the World Asian Middle Eastern or North African White Other
Neurodivergent identity	Autism Attention Deficit Hyperactivity Disorder (ADHD) Dyslexia/Dyscalculia/Dysgraphia Speech/Language disorder Sensory Processing Disorder Tourettes Syndrome Down Syndrome None Other

#### 2.5.2 Depression

The Patient Health Questionnaire Modified for Adolescents (PHQ-A) is a 9 item scale that is used to measure depressive symptoms. Questions are rated on a scale from 0 (“not at all”) to 3 (“nearly every day”). A sum score ranging from 0 to 27 was calculated; scores of 5, 10, 15, and 20 represented mild, moderate, moderately severe, and severe depression, respectively (Johnson et al., 2002).

#### 2.5.3 Anxiety

The Generalized Anxiety Disorder-7 (GAD-7) is a 7 item scale that is used to measure anxiety symptoms. Questions are rated on a scale from 0 (“not at all”) to 3 (“nearly every day”). A sum score, ranging from 0 to 21 was calculated; score cut offs of 5, 10, and 15 represent mild, moderate, and severe anxiety, respectively (Spitzer et al., 2006).

#### 2.5.4 Days of self-harm

Self-harm was measured by asking clients how many days in the 30 days prior to the survey they had engaged in self-harm. Answers could range from 0 to 30.

### 2.6 Data prep

Race, gender, and sexuality questions were condensed due to sample size and to allow for higher powered tests. Race was condensed into 3 levels: Black, White, and Other. Gender was condensed into three levels Male, Female, and Other. Sexuality was condensed into five categories: heterosexual, bisexual, pansexual, homosexual, and other. Neurodivergence was also recorded as a binary variable to indicate if a client held any neurodivergent identity or not.

### 2.7 Data analysis strategy

To identify the rate of overlap between common minority identities and neurodivergence, chi-square tests were conducted between neurodivergence, and race, gender, and sexual orientation.

To assess the effect of holding a neurodivergent identity on mental health symptoms among clients who also hold at least one additional minority identity, a two-way multivariate analysis of variance (MANOVA) was conducted for three different demographic groupings: race, gender, and sexuality. For significant interactions observed in the MANOVA, follow-up two-way analyses of variance (ANOVAs) were performed to further explore the relationships between identities and mental health symptoms. Assumptions for the MANOVA, including linearity, multicollinearity, absence of outliers, multivariate normality, and adequate sample size, were tested and met. However, the assumption of homogeneity of variance–covariance matrices (Box’s M test) was violated for the neurodivergent group’s symptoms at intake. Therefore, the Games-Howell procedure was used for post-hoc comparisons at intake, as it does not assume equal variances. Additionally, a Bonferroni correction was applied to all ANOVAs to control for the increased likelihood of Type I errors due to multiple comparisons.

## 3 Results

### 3.1 Demographics

Chi-square tests of independence were run to analyze the correlation between neurodivergent identity and additional client demographics. The first chi-squared test examined the relationship between gender and neurodivergence, finding a significant association between gender identity and neurodivergent identity x^2^(2, 10,565) = 296.85, *p* < 0.001. Based on descriptive statistics, female-identifying clients are the least likely to identify as neurodivergent; clients with a gender identity that does not fit into the male/female binary are the most likely to identify as neurodivergent (Table 1). A Cramer’s V score was calculated to assess the strength of the association between gender and neurodivergence, finding a small to medium effect size (Cramer’s *V* = 0.169). The next chi-square test examined the relationship between sexual orientation and neurodivergence, finding a significant association between them x^2^(4, 10,279) = 331.59, *p* < 0.001. Clients who identified as straight were the least likely to identify as neurodivergent, while pansexual clients were the most likely to identify as neurodivergent (Table 1). A Cramer’s V score of 0.179 indicates a small to medium effect size between sexual orientation and neurodivergence. Finally, a chi-square test found a significant association between racial identity and neurodivergent identity x^2^(2,10,103) = 159.1, *p* < 0.001, with Black clients significantly less likely to identify as neurodivergent compared to white and other clients (Table 1). A Cramer’s V score of 0.124 indicates a small effect size between racial identity and neurodivergent identity.

**Table 1:** Proportion of respondents who identify as neurodivergent broken down by gender, sexual orientation, and racial identity.

	Neurodivergent	Not neurodivergent
Gender		
Female (*n* = 6,020)	51.15%	48.85%
Male (*n* = 3,267)	57.21%	42.79%
Other (*n* = 1,278)	77.39%	22.61%
Sexual orientation		
Straight (*n* = 4,441)	47.24%	52.56%
Bisexual (*n* = 2,229)	61.51%	38.49%
Pansexual (*n* = 1,005)	73.63%	26.37%
Homosexual/Gay (*n* = 703)	61.59%	38.41%
Other (*n* = 1,901)	62.34%	37.66%
Racial identity		
Black (*n* = 1,214)	44.73%	55.27%
White (*n* = 7,346)	61.90%	38.10%
Other (*n* = 1,543)	51.65%	48.35%

### 3.2 Symptom presentation at intake

#### 3.2.1 Gender

A two-way MANOVA was run to evaluate the effect of gender identity and neurodivergent identity on two dependent variables at intake: depression symptoms and anxiety symptoms. The results of the two-way MANOVA indicated significant main effects of both gender identity (*F*(4, 19,876) = 165.06, *p* < 0.001, Pillai’s Trace = 0.06) and neurodivergent identity (*F*(2, 9,937) = 55.501, *p* < 0.001, Pillai’s Trace = 0.01). Additionally, a significant interaction effect between gender identity and neurodivergent identity was identified (*F*(4, 19,876) = 2.39, *p* = 0.048, Pillai’s Trace = 0.0009), indicating that gender and neurodivergent identities have more than just an additive effect on mental health symptoms.

Follow up two-way ANOVAs were run for depression symptoms and anxiety symptoms to identify where the effect was present. The interaction between gender and neurodivergent identity was significant on depression symptoms (*F*(2, 10,196) = 5.21, *p* = 0.005), but not on anxiety symptoms. As such, a Games-Howell post-hoc test was run to assess the effects of gender identity on each level of neurodivergent identity, and vice versa. For clients who identify as neurotypical, there were significant differences in depression scores between all groupings (*p* < 0.001 for all groups) (Table 2). Neurotypical clients who do not identify as either male or female reported the highest level of depression at intake, with female clients reporting the second highest rates of depression and male clients reporting the lowest rates of depression. Furthermore, significant differences were found between all levels of gender identity for neurodivergent clients (*p* < 0.001 for all groups, Table 3), with the same pattern as neurotypical clients. There are significant differences in depression scores between neurodivergent and neurotypical clients who identify as female and male, respectively. However, there were no significant differences in depression scores between neurodivergent and neurotypical clients who identified as a gender other than male or female (Table 2).

**Table 2:** Differences in mean PHQ9 scores at intake by neurodivergent identity and gender identity.

		Neurodivergent			Neurotypical		
		Female	Male	Other	Female	Male	Other
Neurodivergent	Female	–	2.95****	−1.31****	1.51****	4.65****	−1.30*
	Male	–	–	−4.25****	−1.43****	1.70****	−4.25****
	Other	–	–	–	2.82****	5.96****	0.01
Neurotypical	Female	–	–	–	–	3.14****	−2.81****
	Male	–	–	–	–	–	−5.95****
	Other	–	–	–	–	–	–

*p*-values * < 0.05, ** < 0.01, *** < 0.001, **** < 0.0001. All significant mean differences in bold.

**Table 3:** Differences in mean PHQ9 scores at intake by autistic identity and gender identity.

		Autistic			Non-Autistic		
		Female	Male	Other	Female	Male	Other
Autistic	Female	–	2.02****	−1.46**	1.09**	4.27****	−0.82
	Male	–	–	−3.48****	−0.93	2.25****	−2.84****
	Other	–	–	–	2.55****	5.73****	−0.64
Non-Autistic	Female	–	–	–	–	3.18****	−1.91****
	Male	–	–	–	–	–	−5.09****
	Other	–	–	–	–	–	–

*p*-values * < 0.05, ** < 0.01, *** < 0.001, **** < 0.0001. All significant mean differences in bold.

To further investigate the interactions between gender identity and neurodivergence, two-way MANOVAs were run for both the Autistic and ADHD subpopulations in order to determine if any interactions with gender differ between these two neurodivergent identities. Significant main effects were found for autistic identity (*F*(2, 9,938) = 28.426, *p* < 0.001, Pillai’s Trace = 0.006), along with significant interactions between autism and gender (*F*(4, 19,876) = 2.414, *p* = 0.047, Pillai’s Trace = 0.00097). Similarly, significant main effects were found for ADHD (*F*(2, 9,937) = 42.298, *p* < 0.001, Pillai’s Trace = 0.0086). However, there were no significant interaction effects between ADHD and gender (*F*(4, 19,876) = 1.814, *p* = 0.123, Pillai’s Trace = 0.00073), marking an important distinction between Autistic and ADHD individuals.

As a follow up to the significant interaction effects between gender and autism, two-way ANOVAs were run for depression and anxiety to assess where the interaction effect may be present. After a Bonferroni correction with an updated confidence level of *p* < 0.025, the interaction effect between gender and autism on depression was found to be statistically significant (*F*(2, 10,196) = 5.259, *p* = 0.005), while the effect on anxiety was not (*p* = 0.24). These results are similar to the overall neurodivergent findings. Follow-up Games-Howell tests indicate that significant differences exist between autistic and non-autistic males (*p* < 0.001) and autistic and non-autistic females (*p* < 0.001). There were no significant differences between autistic and non-autistic individuals who hold a gender outside of the gender binary (*p* = 0.09). Additionally, significant differences were present between autistic males and females (*p* < 0.001), autistic female and non-binary clients (*p* = 0.003), and autistic males and non-binary clients (*p* < 0.001). Further details can be found in Table 3.

#### 3.2.2 Sexuality

A two-way MANOVA was conducted to evaluate the effect of sexual orientation and neurodivergent identity on the same two dependent variables previously discussed. Significant main effects were found for both sexual orientation (*F*(8,19,352) = 44.48, *p* < 0.001, Pillai’s Trace = 0.04) and neurodivergent identity (*F*(2, 9,675) = 39.79, *p* < 0.001, Pillai’s Trace = 0.008), as well as in the interaction between the two (*F*(8, 19,352) = 2.75, *p* = 0.005, Pillai’s Trace = 0.002). This indicates that, similar to gender identity, the interaction between sexual orientation and neurodivergent identity has a combined impact on depression and anxiety that is not simply additive.

Follow-up two-way ANOVA tests were run for depression and anxiety to assess where the interaction effect may be present. The interaction effect between sexual orientation and neurodivergent identity was found to be statistically significant on depression (*F*(4, 9,926) = 5.30, *p* < 0.001) and anxiety scores alike (*F*(4, 9,909) = 3.12, *p* = 0.014), and maintained significance after a Bonferroni correction with an updated confidence level of *p* < 0.025 Post-hoc Games-Howell tests were conducted between all levels of gender and neurodivergence for both depression and anxiety symptoms, the results of which are summarized in Tables 4, 5.

**Table 4:** Differences in mean PHQ9 scores at intake by neurodivergent identity and sexual orientation.

		Neurodivergent					Neurotypical				
	Sexual orientation	Straight	Bisexual	Pansexual	Homosexual	Other	Straight	Bisexual	Pansexual	Homosexual	Other
Neurodivergent	Straight	–	−2.16****	−3.40****	−2.63****	−2.12****	0.99***	−1.47****	−3.01****	−1.41	0.40
	Bisexual	–	–	−1.25***	−0.48	0.04	3.15****	0.68	−0.85	0.75	2.56****
	Pansexual	–	–	–	0.77	1.29**	4.40****	1.93****	0.40	1.99**	3.81****
	Homosexual	–	–	–	–	0.52	3.63****	1.16	−0.38	1.22	3.04****
	Other	–	–	–	–	–	3.11****	0.64	−0.89	0.71	2.52****
Neurotypical	Straight	–	–	–	–	–	–	−2.47****	−4.00****	−2.41****	0.59
	Bisexual	–	–	–	–	–	–	–	−1.53*	0.06	1.88****
	Pansexual	–	–	–	–	–	–	–	–	1.60	3.41****
	Homosexual	–	–	–	–	–	–	–	–	–	1.82*
	Other	–	–	–	–	–	–	–	–	–	–

*p*-values * < 0.05, ** < 0.01, *** < 0.001, **** < 0.0001. All significant mean differences in bold.

**Table 5:** Differences in mean GAD7 scores at intake by neurodivergent identity and sexual orientation.

		Neurodivergent					Neurotypical				
		Straight	Bisexual	Pansexual	Homosexual	Other	Straight	Bisexual	Pansexual	Homosexual	Other
Neurodivergent	Straight		−1.92****	−2.46****	−1.77****	−1.47****	0.88***	−0.73	−2.05****	−0.48	0.49
	Bisexual			−0.54	0.15	0.45	2.80****	1.19***	−0.13	1.43*	2.41****
	Pansexual				0.69	0.99*	3.34****	1.73****	0.41	1.97***	2.95****
	Homosexual					0.30	2.65****	1.04	−0.28	1.28	2.26****
	Other						2.35****	0.74	−0.58	0.98	1.96****
Neurotypical	Straight							−1.61****	−2.93****	−1.37*	−0.39
	Bisexual								−1.32*	0.25	1.22**
	Pansexual									1.56	2.54****
	Homosexual										0.98
	Other										

*p*-values * < 0.05, ** < 0.01, *** < 0.001, **** < 0.0001. All significant mean differences in bold.

Building on the investigation done between gender and neurodivergence, additional MANOVAs were conducted to identify any interaction effects between sexual orientation and autism or ADHD. Significant main effects were present for both Autistic (*F*(2, 9,675) = 16.422, *p* < 0.001, Pillai’s Trace = 0.003) and ADHD individuals (*F* (2,9,675) = 22.901, *p* < 0.001, Pillai’s Trace = 0.005). Additionally, both of these neurodivergent identities had significant interactions effects with sexual orientation (Sexuality:Autism *F*(8, 19,352) = 3.397, *p* < 0.001, Pillai’s Trace = 0.003; Sexuality:ADHD *F*(8, 19,352) = 2.262, *p* = 0.0205, Pillai’s Trace = 0.0019).

As such, follow-up two-way ANOVAs were conducted to determine where the interactions were present. Significant interaction effects for both depression and anxiety were present in both the Autistic sample (*F*(4, 9,926) = 5.92, *p* < 0.001 and *F*(4, 9,909) = 3.73, *p* = 0.005 respectively) and ADHD sample (*F*(4, 9,926) = 3.72, *p* = 0.005 and *F*(4, 9,909) = 4.047, *p* = 0.003 respectively). After implementing a Bonferroni correction with an adjusted significance level of *p* < 0.025, all interactions remained significant. Games-Howell tests were run to assess where differences lie between identities. The results of these tests can be found in Tables 6–9.

**Table 6:** Differences in mean PHQ9 scores at intake by autistic identity and sexual orientation.

		Autistic					Non-Autistic				
	Sexual orientation	Straight	Bisexual	Pansexual	Homosexual	Other	Straight	Bisexual	Pansexual	Homosexual	Other
Autistic	Straight	–	−2.31***	−3.18****	−3.66****	−3.03****	0.64	−1.73**	−3.25 ****	−1.66*	−0.51
	Bisexual	–	–	−0.87	−1.34	−0.72	2.95****	0.59	−0.94	0.65	1.80***
	Pansexual	–	–	–	−0.47	0.15	3.82****	1.45*	−0.07	1.52	2.67****
	Homosexual	–	–	–	–	0.62	4.30****	1.93*	0.40	1.99*	3.14****
	Other	–	–	–	–	–	3.67****	1.31*	−0.22	1.37	2.52****
Non-Autistic	Straight	–	–	–	–	–	–	−2.37****	−3.89****	−2.30****	−1.15****
	Bisexual	–	–	–	–	–	–	–	−1.52****	0.7	1.21****
	Pansexual	–	–	–	–	–	–	–	–	1.59***	2.74****
	Homosexual	–	–	–	–	–	–	–	–	–	1.15*
	Other	–	–	–	–	–	–	–	–	–	–

*p*-values * < 0.05, ** < 0.01, *** < 0.001, **** < 0.0001. All significant mean differences in bold.

**Table 7:** Differences in mean PHQ9 scores at intake by ADHD identity and sexual orientation.

		ADHD					Non-ADHD				
	Sexual orientation	Straight	Bisexual	Pansexual	Homosexual	Other	Straight	Bisexual	Pansexual	Homosexual	Other
ADHD	Straight	–	−2.24****	−3.52****	−2.72****	−2.36****	0.74*	−1.71****	−3.16****	−1.82***	−0.344
	Bisexual	–	–	−1.29**	−0.48	−0.13	2.97****	0.52	−0.92	0.41	1.89****
	Pansexual	–	–	–	0.80	1.16	4.46****	1.81****	0.37	1.70**	3.19****
	Homosexual	–	–	–	–	0.36	3.46****	1.01	−0.44	0.90	2.38****
	Other	–	–	–	–	–	3.10****	0.65	−0.8-	0.54	2.02****
Non-ADHD	Straight	–	–	–	–	–	–	−2.45****	−3.90****	−2.56****	−1.08**
	Bisexual	–	–	–	–	–	–	–	−1.45**	−0.11	1.37***
	Pansexual	–	–	–	–	–	–	–	–	1.33	2.82****
	Homosexual	–	–	–	–	–	–	–	–	–	1.48*
	Other	–	–	–	–	–	–	–	–	–	–

*p*-values * < 0.05, ** < 0.01, *** < 0.001, **** < 0.0001. All significant mean differences in bold.

**Table 8:** Differences in mean GAD7 scores at intake by autistic identity and sexual orientation.

		Autistic					Non-Autistic				
	Sexual orientation	Straight	Bisexual	Pansexual	Homosexual	Other	Straight	Bisexual	Pansexual	Homosexual	Other
Autistic	Straight	–	−2.20***	−2.95****	−2.53***	−2.46****	0.23	−1.60***	−2.45****	−1.23	−0.52
	Bisexual	–	–	−0.76	−0.33	−0.26	2.44****	0.59	−0.25	0.97	1.68***
	Pansexual	–	–	–	0.42	0.50	3.18****	1.35**	0.50	1.72**	2.43****
	Homosexual	–	–	–	–	0.07	2.77****	0.92	0.08	1.30	2.01**
	Other	–	–	–	–	–	2.69****	0.85	0.01	1.23*	1.93****
Non-Autistic	Straight	–	–	–	–	–	–	−1.84****	−2.69****	−1.47****	−0.76**
	Bisexual	–	–	–	–	–	–	–	−0.85*	0.37	1.08****
	Pansexual	–	–	–	–	–	–	–	–	1.22*	1.93****
	Homosexual	–	–	–	–	–	–	–	–	–	0.71
	Other	–	–	–	–	–	–	–	–	–	–

*p*-values * < 0.05, ** < 0.01, *** < 0.001, **** < 0.0001. All significant mean differences in bold.

**Table 9:** Differences in mean GAD7 scores at intake by ADHD identity and sexual orientation.

		ADHD					Non-ADHD				
	Sexual orientation	Straight	Bisexual	Pansexual	Homosexual	Other	Straight	Bisexual	Pansexual	Homosexual	Other
ADHD	Straight	–	−1.90****	−2.44****	−1.96*****	−1.78****	0.62	−1.22****	−2.42****	−0.85	−0.03
	Bisexual	–	–	−0.54	−0.06	0.12	2.52****	0.68	−0.52	1.40	1.87****
	Pansexual	–	–	–	0.49	0.66	3.06****	1.22**	0.02	1.59**	2.41****
	Homosexual	–	–	–	–	0.17	2.57****	0.74	−0.46	1.10	1.92****
	Other	–	–	–	–	–	2.40****	0.57	−0.64	0.93	1.75****
Non-ADHD	Straight	–	–	–	–	–	–	−1.84****	−3.04****	−1.47***	−0.65
	Bisexual	–	–	–	–	–	–	–	−1.20**	0.36	1.19***
	Pansexual	–	–	–	–	–	–	–	–	1.56**	2.39****
	Homosexual	–	–	–	–	–	–	–	–	–	0.82
	Other	–	–	–	–	–	–	–	–	–	–

*p*-values * < 0.05, ** < 0.01, *** < 0.001, **** < 0.0001. All significant mean differences in bold.

#### 3.2.3 Race

A two-way MANOVA was conducted to evaluate the effect of racial identity and neurodivergent identity on depression, anxiety, and days of self-harm. While there were significant main effects of racial identity (*F*(4,19,042) = 29.27, *p* < 0.001, Pillai’s Trace = 0.012) and neurodivergent identity (*F*(2, 9,520) = 68.910, *p* < 0.001, Pillai’s Trace = 0.014) on the combined dependent variables, there was no significant interaction between race and neurodivergence. Since this paper focuses on interaction effects between multiple identities, no follow-up tests were conducted for race and neurodivergence.

Finally, two-way MANOVAs were conducted for Autistic and ADHD subpopulations to determine if race interacted with these two neurodivergent conditions differently. As seen in previous tests, both autism and ADHD identities showed significant main effects, however, neither had significant interaction effects with race (*p* = 0.052). As such, no follow-up tests were conducted for these subpopulations.

### 3.3 Treatment outcomes

To assess outcomes post-treatment, the sample was filtered to only include clients who successfully completed treatment and discharged based on clinical recommendation to ensure that we were measuring the effect of a full episode of successful treatment. Of the 9,782 clients who submitted a survey upon discharge, 50.30% routinely discharged, leaving us with a sample size of 4,921 clients. Based on two-way MANOVAs, there are no significant interaction effects between gender and neurodivergence (*F*(2,902) = 1.66, *p* = 0.190, Pillai’s Trace = 0.0037), sexual orientation and neurodivergence (*F*(2, 874) = 2.236, *p* = 0.108, Pillai’s Trace = 0.005), or race and neurodivergence (*F*(2,881) = 2.600, *p* = 0.075, Pillai’s Trace = 0.0059) on the combined dependent variables of depression symptoms and anxiety symptoms by discharge.

## 4 Discussion

### 4.1 Interpretation

The initial investigation revealed disparities in the rate of neurodivergent identification within different marginalized groups. Clients who identified as a racial minority were less likely to identify as neurodivergent compared to their white counterparts, while gender and sexual minority individuals had a higher likelihood of identifying as neurodivergent. These findings align with existing literature (Cooper et al., 2018; Dewinter et al., 2017) and emphasize the importance of recognizing and acknowledging the wide array of identities that an individual holds when implementing treatment.

Furthermore, these differences in rates of identification between groups may indicate societal differences that would benefit from further study. Historically, racial minorities have disparate access to neurodivergent-affirming care such as evaluations and diagnoses, both due to reduced access to healthcare overall (American Medical Association, 2023), and diagnostic tools shaped by white standards (Council of National Psychological Associations for the Advancement of Ethnic Minority Interests, 2016). This limited access to care that can provide context and education around neurodivergent conditions could further reduce an individual’s willingness to identify as neurodivergent therefore reducing the likelihood of identifying as such.

This study also investigated the complex relationship between neurodivergent identity and mental health symptoms within the context of intersecting marginalized identities, intending to fill the gap in the research on neurodivergence and mental health for marginalized communities. Results indicate that neurodivergent identities should not be considered in a vacuum but instead interpreted in conjunction with additional social and personal identities that individuals hold.

A significant interaction between neurodivergence and gender was found in association with depression symptoms, indicating that holding a neurodivergent identity can impact depression symptoms differently depending on an individual’s gender identity. For example, non-binary, neurodivergent individuals reported higher depression scores than their male and female neurodivergent counterparts. This could be because non-binary individuals face unique challenges and pressures from not meeting traditional gender expectations. For example, non-binary individuals are more likely to face discrimination within healthcare settings (Kattari and Hasche, 2016), further increasing pre-existing mental health symptoms. These common situations further emphasize the importance of considering, acknowledging and affirming all identities during the course of mental health treatment.

Additionally, depression symptoms differ for neurodivergent versus neurotypical men and neurodivergent versus neurotypical women, emphasizing the importance of considering both gender and neurodivergent identities when attempting to understand depression scores and how to treat them. As seen by our additional subpopulation tests, Autistic individuals demonstrate a significant interaction effect with gender when it comes to depression. Consider, for instance, an autistic woman who struggles with the sensory input of traditionally feminine clothing such as skirts or heels, but feels pressured to conform to these standards in her traditional office job. The cognitive load of masking her discomfort while in public settings is not distinctly due to her neurodivergence or gender, but a combination of the two identities, and could put her at higher risk of burnout and depression.

Interestingly there was no significant difference found in depression scores between neurotypical and neurodivergent individuals who fall outside of the gender binary. This is potentially due to the heightened prevalence of depression that already exists within the non-binary community, making the neurodivergent identity difference less prevalent.

Sexual orientation and neurodivergent identity also showed significant interactions for both depression scores and anxiety scores. For example, individuals in our study who were both neurodivergent and identified as pansexual or bisexual tend to report higher levels of depression and anxiety when compared to those who were straight, and those who were neurotypical. By taking into consideration an individual’s sexual orientation and their neurodivergent identity, treatment for depression and anxiety can be better personalized to be more impactful.

Finally, no significant interactions were found between racial identity and neurodivergent identity in terms of depression and anxiety scores at intake in this setting. There may be numerous reasons for this. For instance, racial categories used in this analysis may have been too broad. “Black” or “white” as categories can include people with very different life experiences, resources and support systems. This variability could dilute any potential interaction effect between race and neurodivergence. Additionally, while gender and sexual orientation are often up to individual interpretation, racial identity could be considered a cultural and societal construct. As such, racial identities tend to be externally imposed instead of internally constructed. Neurodivergence also tends to be a personal identity up to individual interpretation, and therefore interacts with gender and sexuality internally in a way that has complex interactions on internal states of being like depression and anxiety. Interactions between racial identity and neurodivergence may be more indirect and societally-driven, which could potentially overshadow the direct relationship between the two.

Notably, by discharge, any disparities in mental health symptoms among different identities disappeared. This finding was in contrast to previous literature that demonstrated worse quality of life outcomes for neurodivergent individuals despite higher healthcare utilization rates (Fleming et al., 2017; Loe and Feldman, 2007; Van der Oord et al., 2005), underscoring the importance of holistic and affirming care across all aspects of identity in achieving positive treatment outcomes within mental health settings. When treatment is identity-affirming across multiple aspects of an individual’s identity, treatment outcomes are equitable across the board. This could have multiple underlying reasons. When care is adaptable to multiple aspects of one’s identity, the treatment plans are more tailored to individuals needs. Additionally, when care is identity-affirming, it provides a safe and empowering environment for self-advocacy. It can also create a space where identity-related stress can be reduced, allowing individuals to focus more on underlying issues rather than worry about how others are going to interact with and perceive them.

### 4.2 Limitations and future research

The limitations of this study include the reliance on self-reported data, including neurodivergent status. While self-perception of identity was the important construct for this study, future studies may benefit from considering neurodivergent diagnosis confirmed by medical records. Additionally, researchers and statisticians are still debating the best statistical methods for evaluating intersectionality. Future research should address both of these limitations by developing more robust data collection methods and statistical models that may account for contextual factors such as geographical policies and resources, as well as employing advanced analytical techniques like fuzzy-set theory (Hancock, 2007) to better capture the complexity of intersectional identities.

Additionally, anxiety was measured using a generalized anxiety score and therefore may not have captured nuances of anxiety presentation that may be present between different populations. Future research could broaden the scope of measures to assess differences in anxiety symptoms, as well as other mental health concerns such as substance use or PTSD symptoms.

Mixed methods studies could provide additional context for the interplay between neurodivergence and other marginalized identities that is not available in strictly quantitative research. Allowing individuals who experience the intersections of complex identities would provide a richer understanding of exactly how the identities shape their lives and how they experience these intersections.

Finally, while the sample sizes for ADHD and Autistic clients were large enough to run additional statistical tests on those communities specific larger sample size of neurodivergent individuals who identified as something other than ADHD and Autistic would be beneficial for furthering research on the neurodivergent community as a whole.

## 5 Conclusion

Ultimately, the conclusion of this study echoes the principles of intersectionality at large: health disparities and quality of life are shaped by a complex interplay of various identities. Effective mental health treatment requires viewing a client’s identity holistically; not just as isolated concepts. Furthermore, this study underscores the importance of recognizing neurodivergent identities alongside other social identities, as they contribute greatly to an individual’s sense of self. By affirming a client’s lived experiences and all personal identities, treatment outcomes can be made equitable across a broad population.

## Data Availability

The datasets presented in this article are not readily available because of the sensitive nature of the data. Requests to access the datasets should be directed to elizabeth.kroll@charliehealth.com.
